# Safety of Nonporous Silica Nanoparticles in Human Corneal Endothelial Cells

**DOI:** 10.1038/s41598-017-15247-2

**Published:** 2017-11-06

**Authors:** Ja-Yeon Kim, Joo-Hee Park, Martha Kim, Hyejoong Jeong, Jinkee Hong, Roy S. Chuck, Choul Yong Park

**Affiliations:** 1 Department of Ophthalmology, Dongguk University, Ilsan Hospital, Goyang, South Korea; 2 School of Chemical Engineering and Material Science, Chung-Ang University, Seoul, South Korea; 3 Department of Ophthalmology and Visual Sciences, Montefiore Medical Center, Albert Einstein College of Medicine, Bronx, NY USA

## Abstract

Nonporous silica nanoparticles (SiNPs) are promising drug carrier platforms for intraocular drug delivery. In this study, we investigated the safety of three different sizes of SiNPs (50, 100, and 150 nm) in a human corneal endothelial cell (HCEC) line, B4G12. The HCECs were exposed to different concentrations (0, 25, 50, and 100 µg/ml) of three sizes of SiNPs for up to 48 h. Cellular viability, autophagy, lactate dehydrogenase (LDH) assay, and mammalian target of rapamycin (mTOR) pathway activation were evaluated. Intracellular distribution of the SiNPs was evaluated with transmission electron microscopy (TEM). TEM revealed that the SiNPs were up-taken by the HCECs inside cytoplasmic vacuoles. No mitochondrial structural damage was observed. Both cellular viability and LDH level remained unchanged with up to 100 µg/mL of SiNP treatment. Autophagy showed a significant dose-dependent activation with 50, 100, and 150 nm SiNPs. However, the mTOR activation remained unchanged. Human corneal tissue culture with 100 µg/ml concentrations of SiNPs for 72 h revealed no significant endothelial toxicity. *In vivo* corneal safety of the SiNPs (0.05 ml intracameral injection, 200 mg/ml concentration) was also verified in rabbit models. These findings suggested that 50, 100, and 150 nm SiNPs did not induce acute significant cytotoxicity in corneal endothelial cells at concentrations up to 100 µg/mL. However, long-term toxicity of SiNPs remains unknown.

## Introduction

Human corneal endothelial cells (HCECs) comprise a monolayer of cells located in the innermost area of the cornea. HCECs are important in maintaining corneal transparency through their role in ionic pumping (Na+/K+ pump)^1,2^. They normally do not divide to replace injured cells *in vivo*. Therefore, significant injury or loss of HCECs eventually result in severe corneal edema and loss of transparency^2^.

Amorphous silica nanoparticles (SiNPs) are commonly used as additives to cosmetics, printer toners, packaging, and imaging^3^. They are actively being investigated as promising nanocarrier systems for drug delivery to various human tissues^3^. The large surface area to volume ratio, a stable chemical structure, and ease of surface modification increased the attractiveness of SiNPs as promising drug carrier system^3^. However, biodegradability and biocompatibility of SiNPs should be considered for safer and reliable biologic applications because retaining of SiNPs in human body can cause undesirable long-term health effect. Recently, biodegradable hollow mesoporous types of SiNPs were introduced for more safe effective drug carrier^4,5^.

SiNPs are one of the promising intraocular drug delivery platforms^6^. The recent observation that corneas are permeable to small sizes (5–50 nm) of SiNPs suggests their use as an ophthalmic drug delivery system^7^. However, the possible cytotoxicity is a concern. SiNPs are known to induce biological effects and cellular toxicity depending on the SiNP size, concentration, and cell type^8,9^. Previously, our group reported the effect of various sizes of SiNPs on cultured human corneal epithelial cells and keratocytes. We verified the lack of significant cytotoxicity of 50, 100, and 150 nm SiNPs up to a concentration of 100 µg/ml in culture media^10,11^.

SiNP-based ophthalmic medication can reach intraocular space by topical application or intraocular injection into the anterior chamber or vitreous cavity. Regardless of the route of administration, the major clearance of intraocular SiNP can be through an aqueous outflow pathway^12^. Therefore, a significant contact between SiNPs and HCECs is expected, and the safety issue of HCECs is raised.

To further extend our previous studies, monodisperse nonporous SiNPs with diameters of 50, 100, and 150 nm were employed to investigate how particle size and concentration affect the biological activities of HCECs. Specifically, the effect of different sizes and concentrations of SiNPs on critical biological responses, including cellular viability and autophagy, was evaluated. Furthermore, the effect of SiNPs on the mammalian target of rapamycin (mTOR) pathway, the upstream cellular proliferative pathway, was investigated. Finally, human *ex vivo* corneas and *in vivo* rabbit models were used to verify the safety of SiNPs in corneal endothelial cells.

## Results

### Characterization of SiNPs

The morphologies of each SiNP used in this study were observed by scanning electron microscope (SEM), and the size distribution graphs were previously reported^11^. The average sizes of SiNPs were measured as 50.68, 102.81, and 149.41 nm^11^. Dispersity of the nanoparticles was determined on the basis of the coefficient of variation. Nanoparticles with under a 5% coefficient of variation were defined as monodisperse nanoparticles. The stability of SiNPs in different aqueous solutions was investigated as the measurement of the zeta potential. In distilled water, the SiNPs showed a high zeta potential of over −50 mV and this means good stability and dispersion. In comparison, the SiNPs dispersed in Dubelco’s phosphate-buffered saline (DPBS) showed a lower zeta potential close to a neutral charge. The significant change of zeta potential of the SiNPs means the offset by various salts contained in DPBS. Our result also demonstrated that the charges of the SiNPs are almost neutral in a cell culture medium and this suggested SiNPs are prone to agglomeration in culture medium (Table 1)^10^.

**Table 1:** Size and zeta potential of the SiNPs (at a concentration of 1 mg/mL) investigated in this study.

Size (nm)	Diameter (nm)	Distilled water		DPBS	Media
		Zeta potential (mV)	Dispersity (%)	Zeta potential (mV)	Zeta potential (mV)
50	50.68 ± 2.93	−56.63 ± 3.70	5.79	−3.77 ± 1.36	−1.2 ± 0.6
100	102.81 ± 3.78	−74.67 ± 1.00	3.68	−2.30 ± 1.47	−0.2 ± 0.6
150	149.41 ± 8.39	−75.87 ± 3.20	5.62	−6.90 ± 1.51	−2.7 ± 1.1

*Data presented as mean ± standard deviation. *Abbreviation: DPBS (Dulbecco**’**s phosphate-buffered saline). *Culture medium: DMEM/F12-FBS 10%. (*reprinted with permission from Yim B, Park JH, Jeong H*, *et al*. *The effect of nonporous silica nanoparticles on cultured human keratocytes. IOVS. 2017;58:362–371*.)^10^.

### Intracellular Distribution of SiNPs

**Figure 1:**
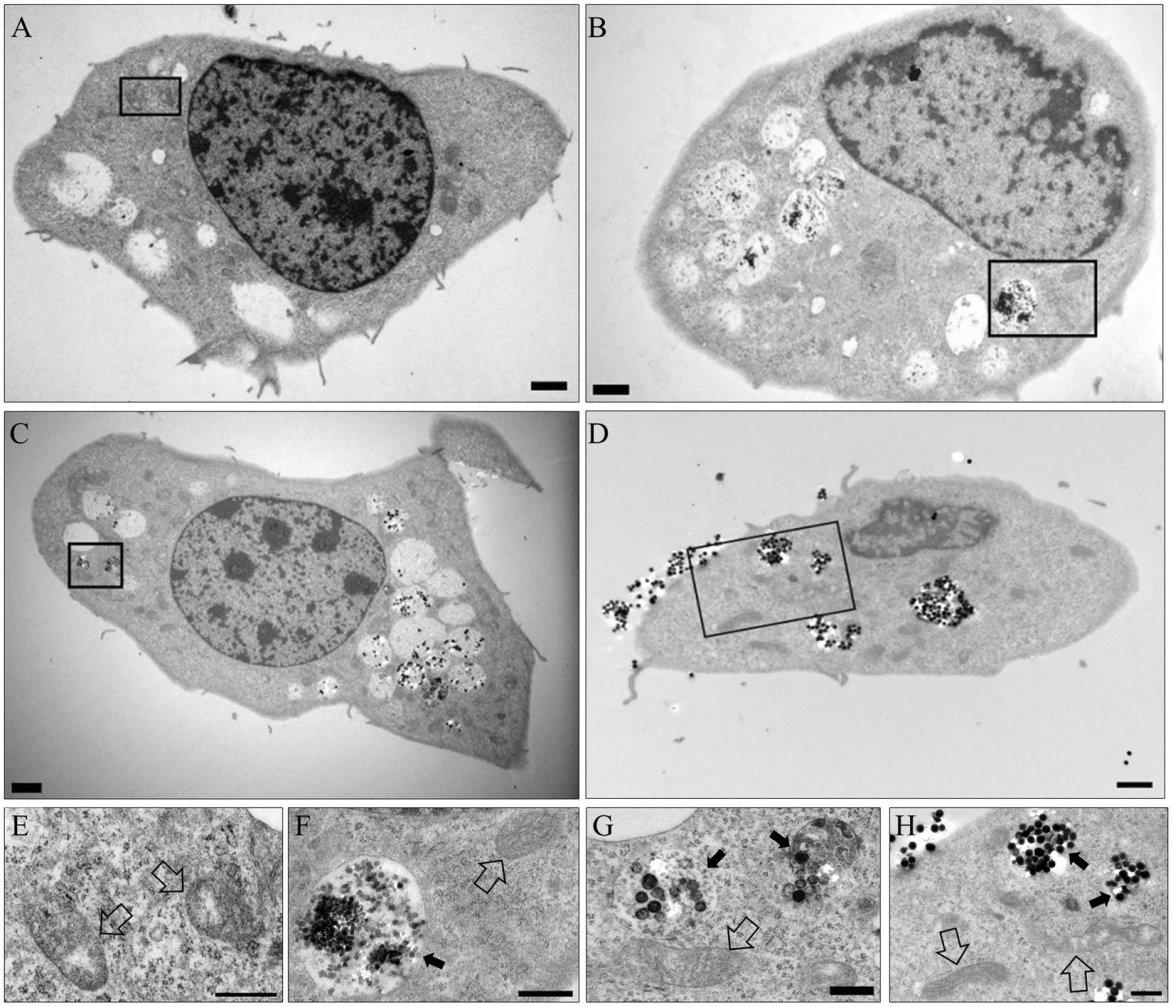
Cellular uptake of SiNPs in HCECs evaluated by TEM. HCECs were cultured with various sizes of SiNPs for 24 h (**B**,**C**, and **D**). The SiNPs (black spheres) were mainly accumulated in the cytoplasmic vesicles (black arrows in **F**,**G**, and **H**), and no SiNPs were observed in the negative control (**A** and **E**). The mitochondria remained intact with no visible damage (blank arrows in E to H). A: negative control with no SiNP, B: 50 nm SiNP added (100 μg/mL), (**C**) 100 nm SiNP added (100 μg/mL), (**D**) 150 nm SiNP added (100 μg/mL). Panels E to H are magnified images of the rectangular area of panels A to D, respectively. Error bar means 2 μm (**A** to **D**) and 1 μm (**E** to **H**).

Transmission electron microscopy (TEM) revealed that SiNPs were localized primarily in cytoplasmic vesicles of HCECs (Fig. 1). No SiNPs were observed inside the mitochondria. Mitochondria and nuclear membrane remained intact with the treatment of SiNPs (Fig. 1).

### Cellular Viability and Lactate Dehydrogenase (LDH) Assay

**Figure 2:**
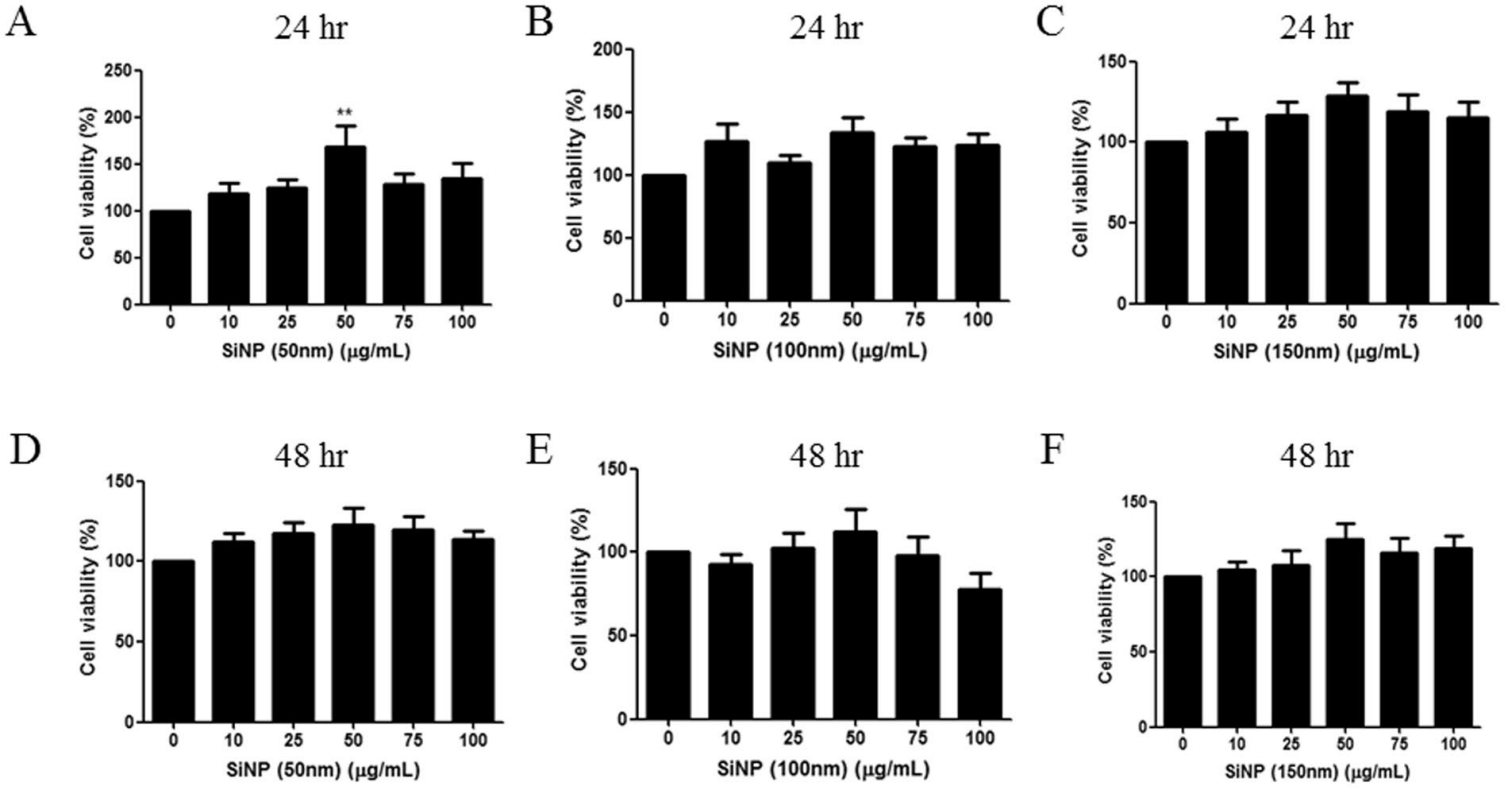
Cellular viability assay. Cellular viability was measured using the CCK-8 kit after incubation at 24 h (**A** to **C**) and 48 h (**D** to **F**) of various concentrations of 50, 100, 150 nm-sized SiNPs. The graphs show a mild increase in viability with SiNP addition for 24 h and 48 h. However, statistical significance was found only with 50 μg/mL of 50 nm-sized SiNPs at 24 h of incubation (**B**). Triplicates of each treatment group were used in each independent experiment. Values are the mean ± SEM from three independent experiments. **p < 0.01.

**Figure 3:**
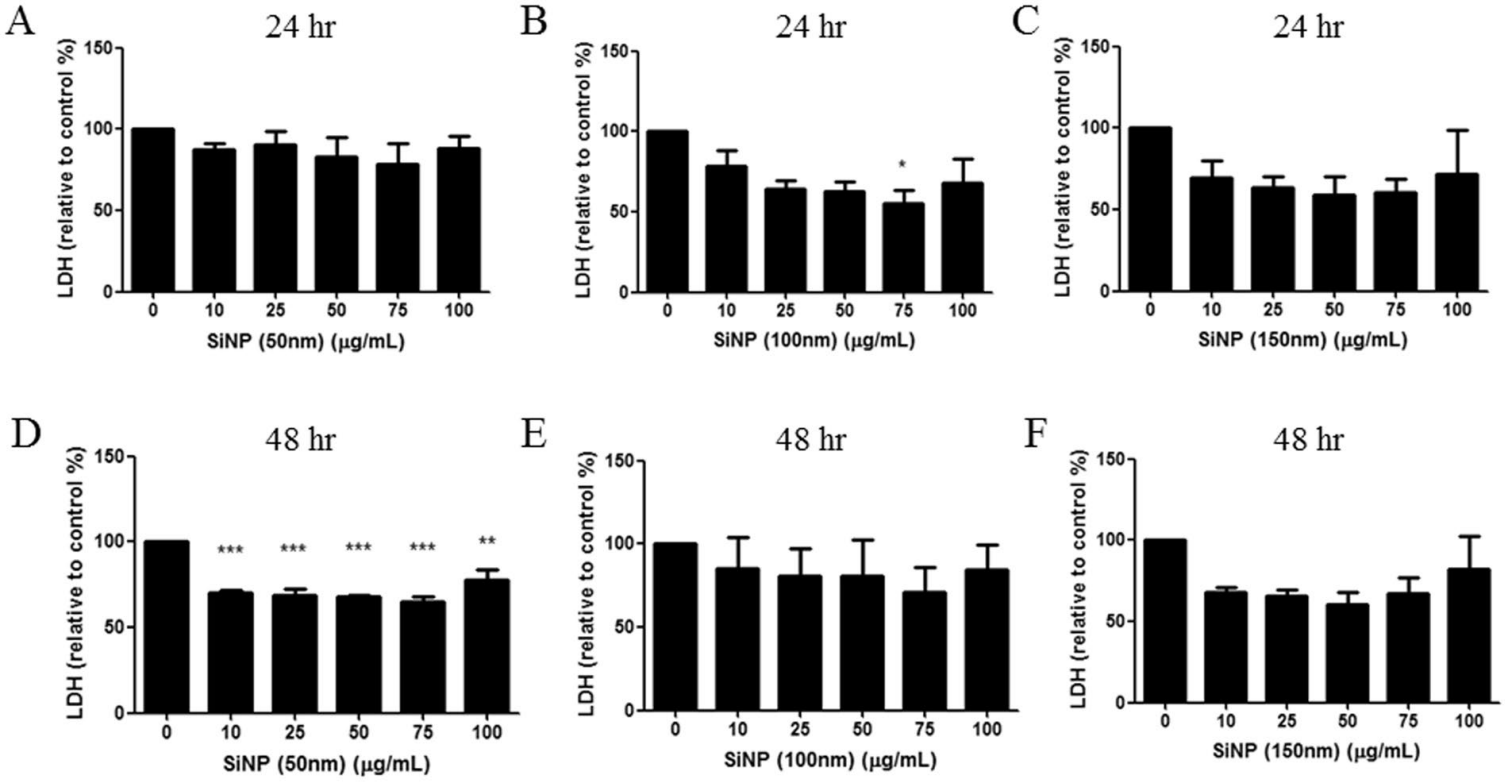
LDH assay. LDH released from HCECs was measured at 24 h (**A** to **C**) and 48 h (**D** to **F**) of incubation of various concentrations of 50, 100, 150 nm-sized SiNPs. LDH decreased significantly with SiNPs of 100 nm at a 75 μg/mL concentration after 24 h of incubation (**B**) and with SiNPs (10–100 μg/mL concentration) of 50 nm after 48 h of incubation. Triplicates of each treatment group were used in each independent experiment. Values are the mean ± SEM from three independent experiments (*p < 0.05, **p < 0.01, ***p < 0.001).

In general, the viability of HCECs did not significantly change because of exposure to SiNPs (Fig. 2). Mild increases in the viability of HCECs were observed sporadically, as shown in Fig. 2, with 50 µg/mL of 50 nm SiNPs after 24 h of incubation. In addition, LDH, which is released through cell membrane damage, showed no increase with the treatment by SiNPs (Fig. 3). A mild decrease in LDH release was specifically observed in 50 nm SiNPs after 48 h of incubation.

### Cellular Autophagy

**Figure 4:**
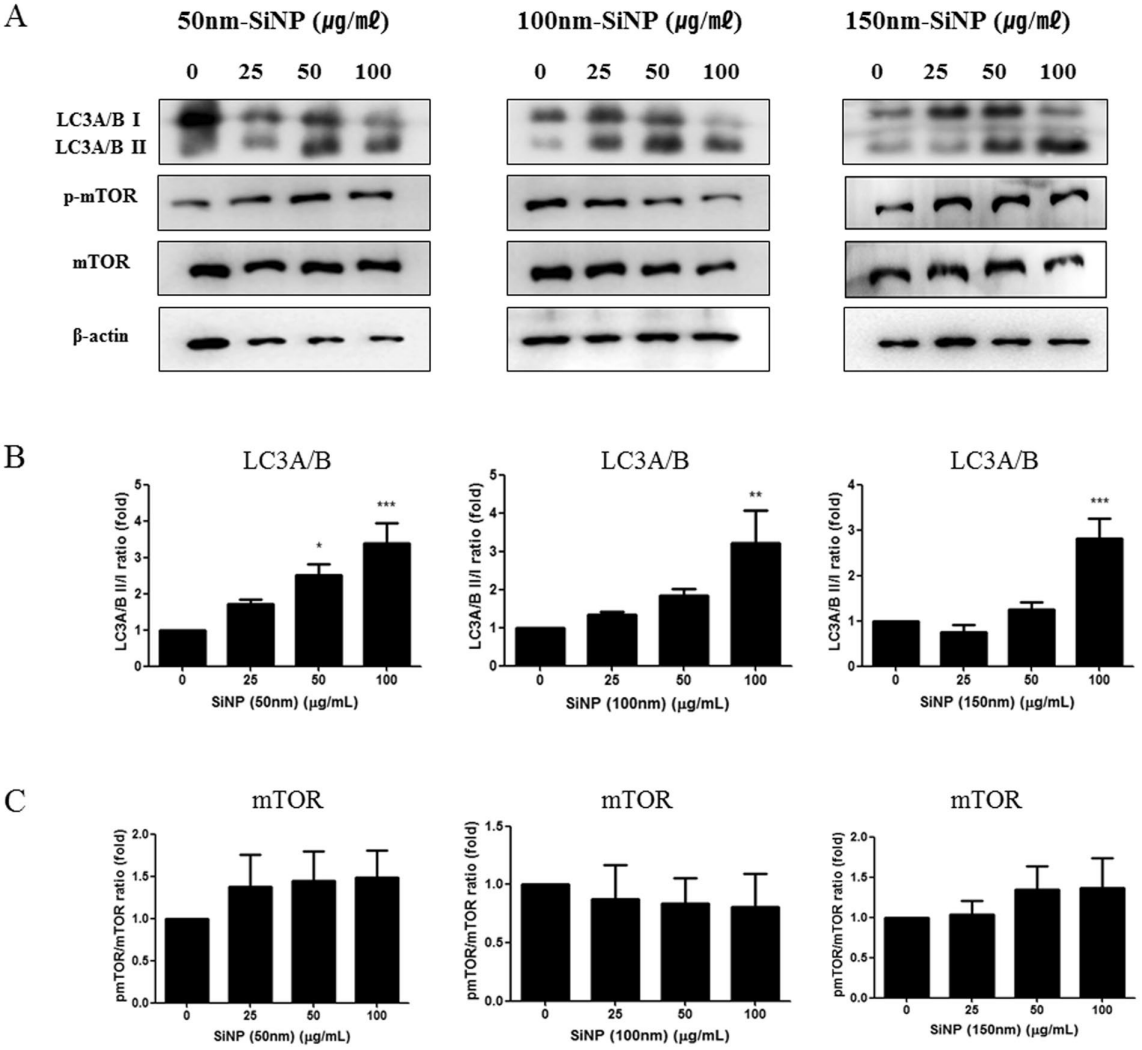
Effect of SiNPs on the autophagy and mTOR pathway of HCECs. (**A**) The expression levels for the autophagy signal, the LC3A/B proteins, and the levels of mTOR were detected by Western blot analysis in HCECs treated with SiNPs for 24 h. For the LC3A/B, the inactive is I form and the active is II form. For the mTOR, the active form is phosphorylated mTOR (p-mTOR). (**B**) The relative densitometric analyses of Western blots showed the dose-dependently increased expression of LC3A/B II form with high concentrations of SiNPs (50, 100, and 150 nm) added. (**C**) The mTOR signal activation did not significantly change with the treatment of SiNPs at a concentration of up to 100 μg/mL. Relative densitometry was calculated as a fold change from the control, and all values (mean ± SEM) were obtained from three independent experiments; each independent experiment was performed in triplicate (*p < 0.05, **p < 0.01, ***p < 0.001).

**Figure 5:**
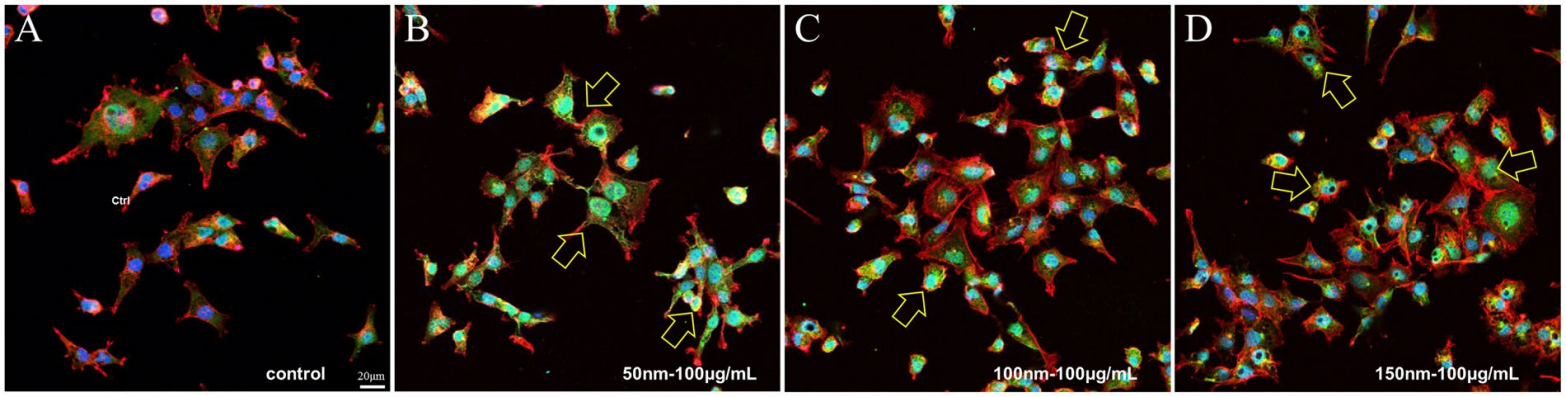
Effect of SiNPs on the autophagy of HCECs. Immunocytochemical staining with LC3B antibody revealed the increased autophagy in HCECs with 100 μg/mL of 50 nm SiNP (**B**), 100 nm SiNP (**C**) and 150 nm SiNP (**D**) addition. Yellow arrows indicate the cells with increased LC3B staining (green). The DAPI stained nucleus with blue and red represents the F-actin. The negative control is HCECs with no SiNP addition (**A**).

The autophagy marker (LC3A/B) was used to investigate the effect of SiNPs on the cellular autophagy system (Fig. 4). With the activation of autophagy, LC3A/B II increased relative to LC3A/B I. The SiNP addition triggered a significant dose-dependent increase in the LC3A/B II protein expression, and this phenomenon was observed in all three sizes of SiNPs, especially at the 100 μg/mL of concentration. The activation of autophagy was also demonstrated by the increased LC3B proteins in the cytoplasm as a result of the SiNPs treatment, and this was shown by the immunocytochemical analysis (Fig. 5).

### mTOR Pathway Activation

We measured the expression level of phosphorylated mTOR (p-mTOR) and mTOR (Fig. 4). The increase in phosphorylated mTOR indicated the activation of the pathway. The expression of p-mTOR showed no significant change with the SiNP addition when compared with the normal control. Our finding suggests that 50, 100, and 150 nm SiNPs do not inhibit mTOR signal transduction, which is one of the most important cell survival pathways.

### ***Ex Vivo*** HCEC Toxicity Assay

**Figure 6:**
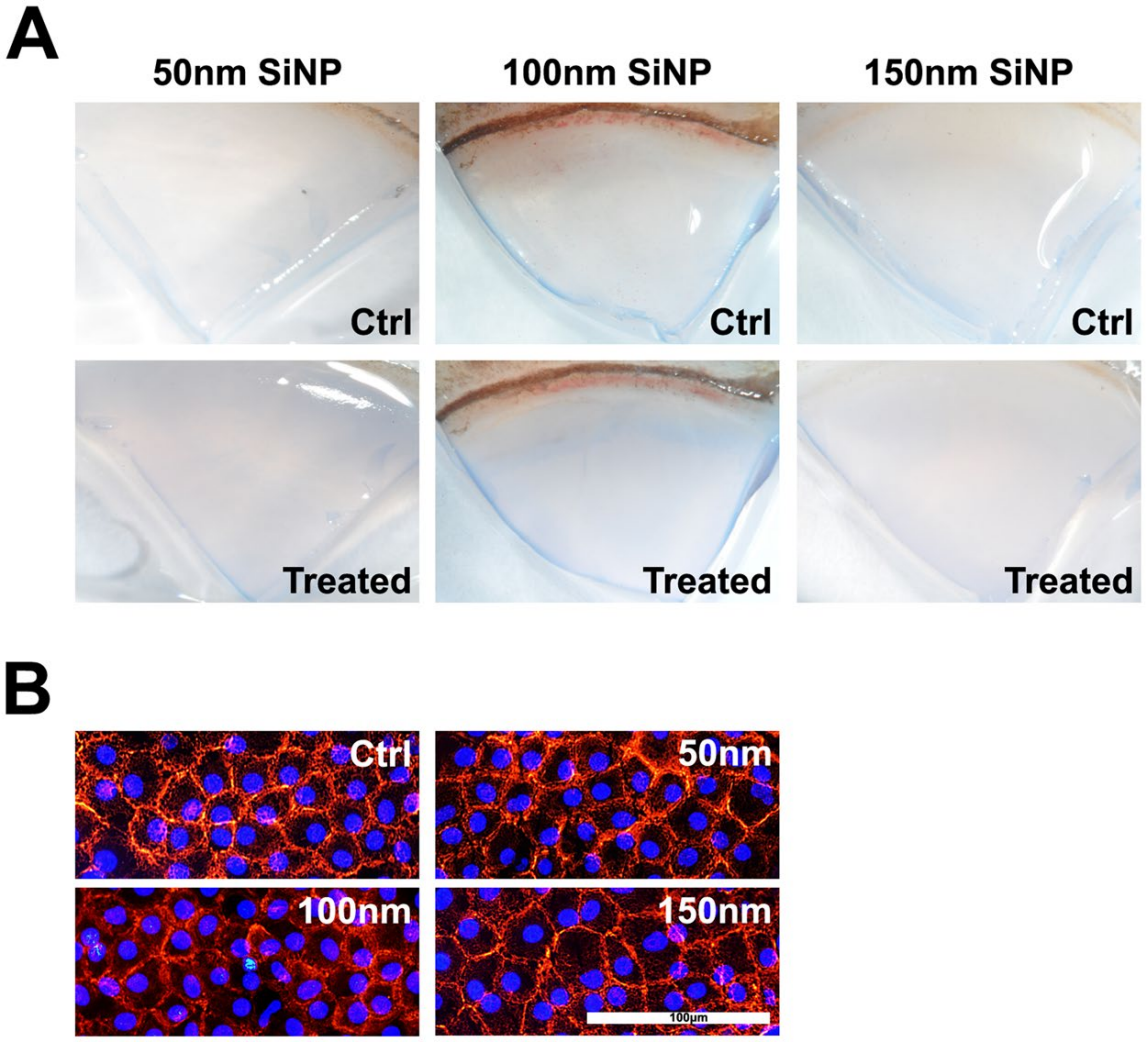
Effect of SiNPs on *ex vivo* human corneas. (**A**) Trypan blue staining of *ex vivo* cornea revealed no significant difference in endothelial cell death (blue stained area) with the addition of 50, 100 and 150 nm-sized SiNPs (100 μg/mL) for 72 h compared with the control (before SiNP treatment). B. The hexagonal architecture of the corneal endothelium is well maintained after 72 h of treatment of each SiNP size. The DAPI stained nucleus with blue and red represents the F-actin.

We used a low concentration of trypan blue (0.005%) mixed with minimum essential media (MEM) to minimize the endothelial cell toxicity during the staining procedure^13^. The trypan blue-stained area showed no significant difference compared with the baseline in all SiNP-treated corneas after 72 h of incubation (Fig. 6). After the SiNP treatment, the corneal endothelial cells effectively maintained a normal hexagonal actin skeleton, as revealed by the phalloidin staining (Fig. 6).

### *In Vivo* Corneal Endothelial Cell Toxicity Assay

**Figure 7:**
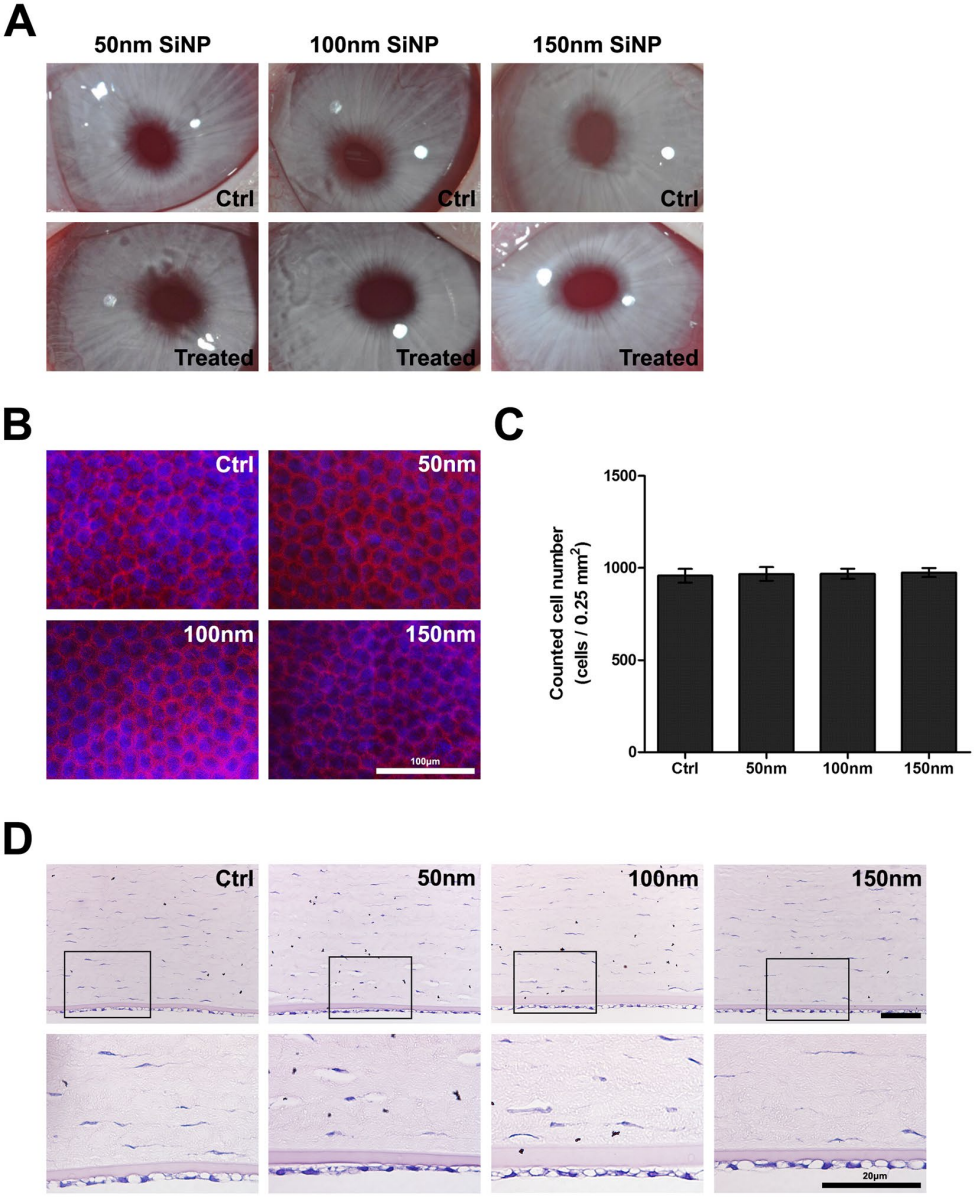
Effect of SiNPs on *in vivo* rabbit corneas. (**A**) Corneal transparency of rabbit corneas is well maintained at two weeks after the intracameral injection (200 mg/mL, 0.05 ml) of three sizes (50, 100, and 150 nm) of SiNPs. (**B**) The hexagonal architecture of the corneal endothelium is well maintained after two weeks of treatment of each SiNP size. The DAPI stained nucleus with blue and red represents the F-actin. (**C**) Endothelial cell count was performed in 500 μm × 500 μm square areas. Cell density was not affected by two weeks after SiNPs treatment. (**D**) Histologic examination (Hematoxylin and Eosin staining) revealed normal corneal endothelial cell layer in both control and all treated groups. Lower panels are magnified images of rectangular area of upper panels.

The rabbit corneas maintained their transparency for two weeks after the intracameral injection of SiNPs. No corneal haze, edema, or limbal vascular abnormality was observed both in the control and in the SiNP-treated eyes. The iris appeared normal, and the crystalline lens was clear both in the control and in the SiNP-treated eyes. The hexagonal structure of corneal endothelium was well maintained, and no significant difference was found in the endothelial cell density between the control and treated groups (Fig. 7). The histopathologic evaluation revealed no difference in the corneal endothelial appearance between SiNPs-treated and control corneas (Fig. 7).

## Discussion

In this study, we found that monodisperse nonporous SiNPs of 50, 100, and 150 nm sizes induced no significant cytotoxicity in cultured HCECs up to 48 h and at a concentration of up to 100 µg/mL. Cellular autophagy showed a dose-dependent increase with high concentrations of SiNPs, but the mTOR pathway remained intact. The human corneal tissue culture model demonstrated no significant cytotoxicity of SiNPs (100 µg/mL) after 72 h of exposure. The safety of SiNPs was further verified *in vivo* with the rabbit intracameral injection model.

For efficient intraocular drug delivery, nanoparticle-based ocular drug delivery systems, including gold, carbon, lipid, chitosan, liquid crystalline, and albumin nanoparticles, have been actively investigated^14–19^. As a nanocarrier system, SiNPs have a great potential for ocular drug delivery^20,21^. SiNPs have a stable chemical structure, a large surface area to volume ratio, ease of surface modification, and tolerable biodegradability, which all increase their attractiveness for biologic applications^3^. However, safety must be verified^22^, and as mentioned earlier, the safety of SiNPs has always been a challenge for clinical application. The crystalline form of silica is toxic to alveolar cells and can induce irreversible pulmonary silicosis^23,24^. Conversely, amorphous silica, such as nonporous silica particle, is considered relatively biologically safe because of its biodegradability^25–27^. Biodegradation and clearance are essential for biomedical use of nanoparticles because chronic accumulation of nanoparticles in human bodies can lead to serious long term complications. It was reported that 5 to 10 nm sized nonporous SiNPs degraded in two weeks^28^. Furthermore, smaller and porous SiNPs degraded faster than larger and nonporous SiNPs^28^. It was reported that mesoporous SiNPs biodegraded in human umbilical vein endothelial cells and human embro kidney (HEK) 293 T cells^29,30^. Nevertheless, negative reports have suggested the significant nanotoxicity caused by SiNPs^31–35^. The nanotoxicity of SiNPs seems to be dependent on size, dose, and cell types^9,36^. Therefore, the toxicity of silica nanoparticles should be evaluated independently in every target organ system by means of both *in vivo* and *in vitro* toxicity tests. For the ocular system, no direct toxicity of SiNPs in retinal and corneal cells has been previously reported^6,37^. In addition, our group recently reported the *in vitro* safety of 50, 100, and 150 nm-sized nonporous SiNPs by using the primary culture of human corneal epithelium and keratocytes^10,11^.

Although, the lack of acute toxicity of SiNPs on cultured human corneal epithelial cells and keratocytes was verified in our previous reports^10,11^, further verification of the safety of SiNPs in HCECs is madatory. Corneal epithelial cells and keratocytes possess regenerative capability and they can actively repopulate the damaged cells within several weeks after the injury. However, HCECs are lack of regenerative potential and any injury to HCECs can lead to the irreversible loss of corneal transparency and serious visual deterioration^2^. After penetrating the cornea, all nanoparticles disperse into aqueous humor. The aqueous outflow pathway is the most important clearance pathway for intraocular porous silicon micro-particles^12^. Until full clearance is obtained through the trabecular meshwork, SiNPs can remain in contact with HCECs for a prolonged time. Therefore, our current study regarding HCEC safety is an important step in the safety clearance for the future development of SiNPs as an ocular topical drug delivery system.

In this study, SiNPs treatment increased autophagy in HCECs. This finding is consistent with previous reports that SiNPs can induce cellular stress^38–41^. The similar elevation of autophagy by SiNPs was observed in our previous reports using human corneal epithelial cells and keratocytes^10,11^. Autophagy is known as a natural cellular process that cleans up unnecessary and dysfunctional cellular components for recycling^42^. It also helps cells to overcome external stress and survive in harsh environments. Recently, it was reported that autophagy promotes the degradation of polyethyleneimine-alginate nanoparticles in endothelial progenitor cells^43^. Another study reported that the increased osteoblast proliferation was induced by autophagy after tantalum nanoparticle exposure^44^. Therefore we hypothesize autophagy system is one of the cellular mechanism to overcome SiNP-induced stress. It is noteworthy that mTOR pathway and cell viability remained intact even with increased autophagy in our study.

Unfortunately, the primary culture of HCECs has a limited proliferation capacity. Using primary cultured HCECs from different donors can cause donor-specific effects, such as age and topography-related (central vs. peripheral origin) effects on experimental results^2^. Therefore, because our studies required a great number of homogeneous cells, we used the HCECs cell line B4G12 in this study. This cell line is one of the two clonal lines obtained from the HCEC-12 cell line, which was made by the immortalization of parental cells from a 91-year-old woman (http://www.creative-bioarray.com/HCEC-B4G12-CSC-C3457-item-1468.htm). B4G12 can provide sufficient homogenous cell numbers for multiple experiments. As observed in many cell lines, the properties of HCECs and B4G12 are not always the same. Nevertheless, B4G12 adequately expresses HCEC-specific 9.3. E-antigen, ZO-1, and occludin^45^. In addition, B4G12 transplanted to replace corneal endothelium in rabbit eyes was able to successfully restore the Na+/K+ pump function and maintain corneal transparency^46^. B4G12 has been actively used as an ideal model for differentiated HCECs because of its morphologic and functional similarity^47–49^.

To compensate for the limitation of using immortalized B4G12 cells, we further verified the safety of SiNPs by using *ex vivo* human corneas and an *in vivo* rabbit model. We found that 50, 100, and 150 nm-sized SiNPs induced no significant endothelial toxicity in both human and rabbit corneas. The well-maintained hexagonal architecture suggests that the SiNPs did not cause significant cellular damage at the tested concentration.

Our study has several limitations. First, we tested limited sizes and concentrations of SiNPs (up to 100 µg/mL and three different sizes). A further increase in SiNP concentration to a higher range or with smaller particle sizes may possibly induce significant cellular damage in HCECs. Moreover, the uptake of SiNPs by cells may eventually lead to the chronic perturbation of intracellular mechanisms and cellular damage. Therefore, a close *in vivo* observation for an extended period of time (e.g., years) is necessary to further verify the safety of SiNPs. Second, the long-term exposure effects of SiNPs on HCECs are lacking. Note that *ex vivo* human corneal tissue culture is different from the *in vivo* environment because the dynamic circulation of aqueous humor is absent in a tissue culture setting.

In summary, we found SiNPs of sizes 50, 100, and 150 nm to be safe in HCECs at up to 72 h of exposure. The cellular uptake of SiNPs was localized inside the vacuoles of the cytoplasm with no nuclear membrane or mitochondrial damage. The cellular survival in the mTOR pathway remained intact, although some activation of autophagy was observed at higher concentrations of SiNPs. The cellular viability of HCECs was not affected at up to 48 h of exposure to SiNPs at the tested concentrations. The human corneal tissue culture revealed no significant toxicity of SiNPs at 100 μg/mL of concentration at up to 72 h. The *in vivo* rabbit model further verified the lack of toxicity of SiNPs in corneal endothelial cells.

## Conclusions

Our current study verified the safety of 0, 100, 150 nm sized SiNPs in human HCECs. These findings can facilitate the future development of the SiNP-based ophthalmic drug delivery method for intractable eye diseases.

## Materials and Methods

### Synthesis and Characterization of Nanoparticles

Three sizes of silica (SiO_2_) nanoparticles (SiNPs; sizes: 50, 100, and 150 nm) were manufactured using the Stöber synthesis method as previously reported^10,11^. The synthesis of 50 nm of SiNPs started with mixing 2 mL of ammonia (NH_4_OH, 28%, Junsei, Tokyo, Japan) and 50 mL of ethyl alcohol (EtOH, anhydrous, 99.5%, Daejung, Kyeonggi, Korea) were first mixed. Then, 1 mL of tetraethyl orthosilicate (TEOS, Samchun, Gyunggi, Korea) was added. Similarly, 100 nm and 150 nm of SiNPs were prepared using equal molar ratios of ingredients. Afterward, 1.5 mL of TEOS was added to the mixture of 3 mL solution of ammonia in 50 mL of ethyl alcohol. Then, the solutions were stirred continuously for 12 h at an ambient condition (25 °C, 1 atm). Smaller-sized SiNPs could be produced by quickly adding TEOS while stirring the solution. The prepared SiNPs were washed with EtOH three times using centrifugation (10,000 rpm, 15 min). The final SiNP precipitates were dispersed in distilled water.

The surface charge of the prepared SiNPs was measured by the zeta potential (SZ-100, Horiba, Kyoto, Japan) in both distilled water and DPBS. In addition, SEM (SIGMA, Carl Zeiss, Oberkochen, Germany) images and ImageJ software were used to analyze the size and distribution of SiNPs. The dispersity of SiNPs was defined as the coefficient of variation (Dispersity (%) = ${\rm{\sigma }}/d\times 100$, where ${\rm{\sigma }}$ is the standard deviation and *d* is the mean size)^50^.

### Cell Culture

The limited proliferative capability of cultured primary HCECs makes any *in vitro* experiment requiring large numbers of these cells unrealistic. Therefore, we used an established HCEC line (i.e., B4G12 cells) in this study. B4G12 cells (Cat no.CSC-C3457-CRA, Creative Bioarray, Shirley, NY, USA) were used. The cells were cultured in tissue culture-treated plastic at 4 × 10^4^ cells/cm^2^. The culture medium recommended by the company, which contains human endothelial serum free medium (Cat no. CM-345L7, Creative Bioarray, Shirley, NY, USA) and 10 ng/ml of fibroblast growth factor-2 (Cat no. CSC-CTK0134, Creative Bioarray, Shirley, NY, USA), was used. After reaching confluency, the cells were harvested and resuspended in the culture medium. The cells were plated in 75 cm^2^ tissue flasks and then maintained at 37 °C in a 5% CO_2_ and 95% air-humidified atmosphere. The culture medium was changed every three days, and the cells were passaged using 0.25% Trypsin- ethylenediaminetetraacetic acid (EDTA) (Gibco BRL, Carlsbad, CA, USA). The passage number ≤5 was used for the study.

### Treatment of SiNPs

SiNPs with sizes of 50, 100, and 150 nm were confirmed using SEM. Before being mixed into the HCECs culture medium, the stock solution of the SiNPs, which was 10 mg/mL in DPBS (Gibco), were sonicated for 30 min. HCECs were cultured in the SiNP-containing culture medium in a 5% CO_2_ and 95% air-humidified atmosphere at 37 °C for 24 h or 48 h.

### Electron Microscopy Analysis

The intracellular distribution of SiNPs in HCECs was investigated by TEM as previously described^11^. The HCECs were treated with three sizes of SiNPs (100 µg/ml) for 24 h, and then the cells were fixed in 3.7% paraformaldehyde (Sigma–Aldrich, St. Louis, MO, USA) and 2.5% glutaraldehyde (Sigma–Aldrich, St. Louis, MO, USA) in a 0.1 M phosphate buffer (PB; pH7.6) overnight. After washing in 0.1 M PB, the HCECs were fixed in 1% osmium tetroxide in the same buffer for 1 h. Dehydration of cells was performed with a series of graded EtOH (Merck, Kenilworth, NJ, USA), and then the cells were embedded in an epoxy embedding medium (Sigma–Aldrich, St. Louis, MO, USA). Polymerization was then performed at 60 °C for three days. Ultrathin sections (60–70 nm) of the samples were obtained by an ultramicrotome (Leica Ultracut UCT, Leica, Germany). Obtained sections collected on grids (200 mesh) were examined under the TEM (JEM-1010; JEOL, Tokyo, Japan) operating at 60 kV. The images were recorded by a charge-coupled device camera (SC1000; Gatan, Warrendale, PA, USA). Length on the electron micrograph was measured using the GMS software (Gatan, Warrendale, PA, USA). The normal control for TEM was incubated in a corneal basal medium without SiNPs for 24 h.

### LDH Assay

Necrotic cell death with plasma membrane damage was evaluated using an LDH cytotoxicity detection kit (Takara Bio Inc., Shiga, Japan)^10^. The experimental procedure was previously described and performed following the manufacturer’s protocol^11^. Briefly, HCECs were cultured at 3 × 10^3^ cells/well in a 96-well plate and incubated for 24 h and 48 h. Following the adherence of cells, 50, 100, and 150 nm SiNPs were applied to the cells for 48 h dose dependently at 0, 25, 50, and 100 µg/ml. The wells with no SiNP addition and the wells with 1% triton X-100 addition were used as the negative and positive controls, respectively. Following the incubation of cells, cell free supernatants were transferred into a new 96-well plate. The wells were incubated with the reaction mixture for 20 min at room temperature (RT). Absorbance at 490 nm was measured.

### Cell Viability Assay

A commercial cell counting kit (CCK-8; Dojindo Molecular Technologies, Inc., Kumamoto, Japan) was used to measure HCECs’s viability according to the manufacturer’s protocol^11^. Briefly, the HCECs were cultured at 3 × 10^3^ cells/well in a 96-well plate and incubated for 24 h. Following the adherence of cells, 50, 100, and 150 nm SiNPs were added to the culture media for 24 h and 48 h over a range of concentrations (0, 25, 50, and 100 µg/ml). After the appropriate incubation, 10 µL of CCK-8 solution was added to each cultured well, and absorbance at 450 nm was determined after 2 h of incubation of the HCECs with the reagent at 37 °C.

### Western Blot Analysis

Western blot analysis was performed following the previously reported method^11^. All SiNP-treated HCECs were lysed in an ice-cold radioimmunoprecipitation assay buffer [50 mM Tris-HCl (pH 8.0), 150 mM NaCl, 1% NP-40, 0.5% deoxycholate, and 0.1% sodium dodecyl sulfate (SDS)] for 30 min. Debris was removed by centrifugation at 16,000 g for 1 min. Equal amounts (20 μg) of total cell protein were separated by SDS–polyacrylamide gel electrophoresis and transferred to a polyvinylidene difuoride membrane. After blocking with 5% bovine serum albumin (BSA) in tris buffered saline with tween 20 (10 mM Tris, pH 8.0, 150 mM NaCl, 0.1% Tween 20) for 1 h at RT, the membranes were incubated overnight at 4 °C with the following primary antibodies: rabbit anti-phospho-mTOR (1:1000; catalog number: 5536; Cell Signaling), rabbit anti-mTOR (1:1000; catalog number: 2983; Cell Signaling), rabbit anti-LC3A/B (1:1000; catalog number: 12741; Cell Signaling, Beverly, MA, USA), and mouse anti-β-actin (1:10,000; catalog number: sc-47778; Santa Cruz, Biotechnology, Dallas, Texas, USA). Then, incubation with peroxidase-conjugated secondary antibody was performed for 1 h at RT. Blots were developed using an enhanced chemiluminescence kit (catalog number: RPN2232; GE healthcare, Buckinghamshire, UK) and visualized using a Fujifilm Image Reader LAS-3000 (Fujifilm, Tokyo, Japan). Densitometric analysis was performed using Multi Gauge V3.0 (Fujifilm Life Science, Tokyo, Japan). Each experiment was repeated at least as triplicate.

### Immunocytochemistry

As previously reported^11^, the HCECs were seeded at a density of 2 × 10^4^ cells per milliliter and grown on 4-well Lab-Tek chamber slides (Nalgene Nunc International, Penfield, NY, USA), and 100 µg/mL of 50, 100, 150 nm-sized SiNPs was treated for 24 h. The HCECs were fixed with 3.7% paraformaldehyde for 10 min at RT, and permeabilization was performed using 0.1% triton x-100 for 5 min at RT^11^. After washing with DPBS, the blocking of non-specific antigen site was done using 1% BSA in DPBS for 30 min at RT. The chamber slides were incubated at 4 °C overnight with rabbit polyclonal anti-LC3B (0.5 ug/mL; catalog number: L10382; Molecular Probes, ThermoFisher Scientific Inc., Waltham, MA, USA). Next, the slides were washed with DPBS and incubated with Alexa 488-conjugated donkey anti-rabbit antibody (1:1000; catalog number: A21206; Molecular Probes) for 2 h at RT. Tetramethylrhodamine isothiocyanate (TRITC)-conjugated phalloidin (1 µg/mL; Sigma–Aldrich, St. Louis, MO, USA) was used for staining of F-actin. Nuclear counterstaining was performed using 4′,6-diamidino-2′-phenylindole (DAPI, catalog number: P36931; Molecular Probes) with a mounting solution. Finally, slides were examined under a fluorescence microscope.

### *Ex Vivo* HCEC Toxicity Assay

Untransplantable human corneal donor buttons (“research use only” grade) were provided by Eversight International (Seoul, South Korea). Two corneas were from a 63-year-old male donor and another two corneas were from a 59-year-old male donor. Informed consent regarding the possible research use of the corneas was obtained from the donors when they agreed on tissue donation form. The experiment followed the tenets of the Declaration of Helsinki and was approved by the institutional review board of Dongguk University, Ilsan Hospital, Goyang, South Korea. The corneas were harvested at the date of death of the donors and used for the experiment at 9 and 11 days after death. Fresh human corneal donor buttons (n = 4) were divided by quadrant segments. Each segment was stained with 0.005% trypan blue mixed with minimum essential medium (MEM) for 5 min. Corneal endothelial cell viability was assessed by examining the blue-stained area under an inverted-phase contrast microscope. After the baseline viability assessment, the corneal segments were divided into four groups (n = 4 in each group), and each segment was incubated at 37 °C in a 5% CO_2_ and 95% air-humidified atmosphere for 72 h. The tissue culture medium was serum-free MEM containing L-glutamine (2 mM), NaHCO3 (20 g/L), penicillin (100 IE/mL), and streptomycin (0.1 mg/mL). Three different sizes (50, 100, and 150 nm) of SiNPs were mixed in the tissue culture medium at a 100 µg /mL concentration (n = 4 each group). The tissue culture medium with no SiNPs was used for the control group (n = 4). After 72 h of incubation with SiNPs, the corneal segments were stained again with 0.005% trypan blue mixed with MEM for 5 min. Any increase in the blue-stained area from the baseline was used as the indicator of corneal endothelial cell toxicity, and these were compared among the groups. After trypan blue evaluation, the corneal segments (n = 3 each group) were fixed in 10% formalin and incubated with TRITC-conjugated phalloidin (1 µg/mL; Sigma–Aldrich). The tissue was rinsed three times with PBS (5 min per rinse), and the whole-mount corneas were mounted endothelial-side down on a slide and stained with DAPI (catalog number: P36931; Molecular Probes). The slides were examined with a fluorescent microscope.

### *In Vivo* Rabbit Corneal Endothelial Cell Toxicity Assay

To investigate the *in vivo* corneal effect of SiNPs, we used 12 New Zealand white rabbits (males, weighing 2.5–3.0 kg). The animals were treated in compliance with the ARVO Statement for the Use of Animals in Ophthalmic and Vision Research. Experimental protocol was approved by Institutional Animal Care and Use Committee of Dongguk University, Ilsan Hospital (reference number: 2016–03146). The rabbits were divided into four groups (n = 3, each group), including the negative control group, and treated with intracameral injection of the SiNP solution (0.05 ml) of each size of SiNP (50, 100, 150 nm-sized SiNPs, 200 μg/mL concentration mixed with balanced salt solution) to the right eyes. To consider the average aqueous humor volume of rabbits (290 μL), the final concentration of SiNP in the anterior chamber was estimated as 30 μg/mL. The left eyes were used for the negative control. Corneal photographs of both eyes were taken at baseline, days 1, 2, and 3, and weeks 1 and 2. Corneal transparency was evaluated according to these photographs. At two weeks after the intracameral injection, the rabbits were euthanized and both eyes were enucleated. Tissues were fixed in 10% formalin, and a histopathologic examination using hematoxylin and eosin staining was performed. One cornea from each group was incubated with TRITC-conjugated phalloidin (1 µg/mL; Sigma–Aldrich). The tissue was rinsed three times with PBS (5 min per rinse), and the whole-mount corneas were mounted endothelial-side down on a slide and stained with DAPI (catalog number: P36931; Molecular Probes). The slides were examined with a fluorescent microscope. Using the fluorescent pictures, endothelial cell count (DAPI stained nucleus) in three 500 μm × 500 μm square areas was calculated in each cornea using ImageJ software (http://imagej.nih.gov/ij/).

### Statistical Analysis

Data were presented as mean ± standard error, and statistical significance was determined by ANOVA and Dunnett’s multiple comparison test. *P* values of less than 0.05 were regarded as significant by GraphPad Prism Ver. 5.01 (GraphPad Software Inc., La Jolla, CA, USA).

## Electronic supplementary material

supplementary material
