# Supplementary material for: Safety of Nonporous Silica Nanoparticles in Human Corneal Endothelial Cells
Source: Sci Rep. 2017 Nov 6;7:14566. doi: 10.1038/s41598-017-15247-2 (PMC5674045; doi:10.1038/s41598-017-15247-2)

Safety of Nonporous Silica Nanoparticles in Human Corneal Endothelial Cells

Ja-Yeon **Kim** MS[a], Joo-Hee **Park** PhD[a], Martha **Kim** MD[a], Hyejoong **Jeong** BS[b], Jinkee **Hong** PhD[b], Roy S. **Chuck** MD, PhD[c], Choul Yong **Park** MD, PhD[a]

a. Department of Ophthalmology, Dongguk University, Ilsan Hospital, Goyang, South Korea
b. School of Chemical Engineering and Material Science, Chung-Ang University, Seoul, South Korea
c. Department of Ophthalmology and Visual Sciences, Montefiore Medical Center, Albert Einstein College of Medicine, Bronx, NY, USA

Supplementary material

Full length gel of electrophoresis

Figure 5.

p-mTOR

50 nm SiNP and 100 nm SiNPs

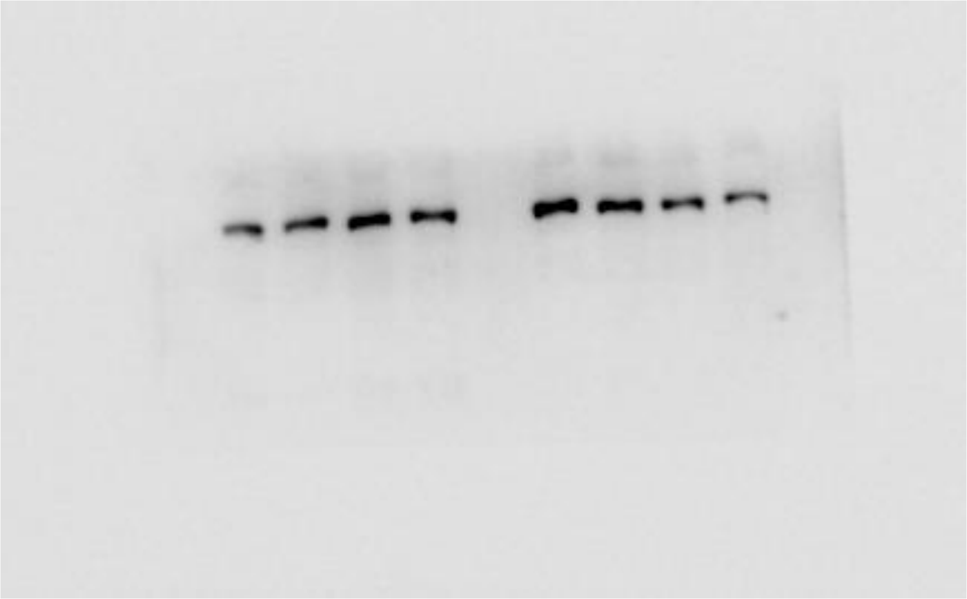

150 nm SiNP

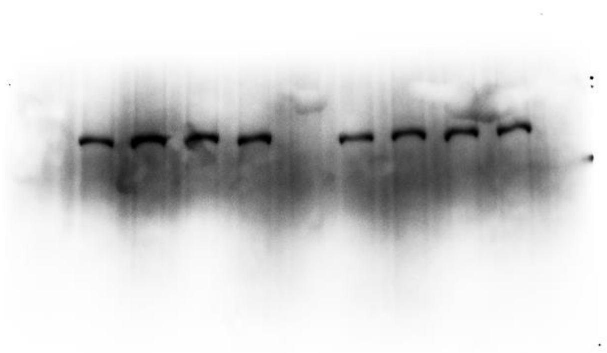

mTOR

50 nm SiNP and 100 nm SiNPs

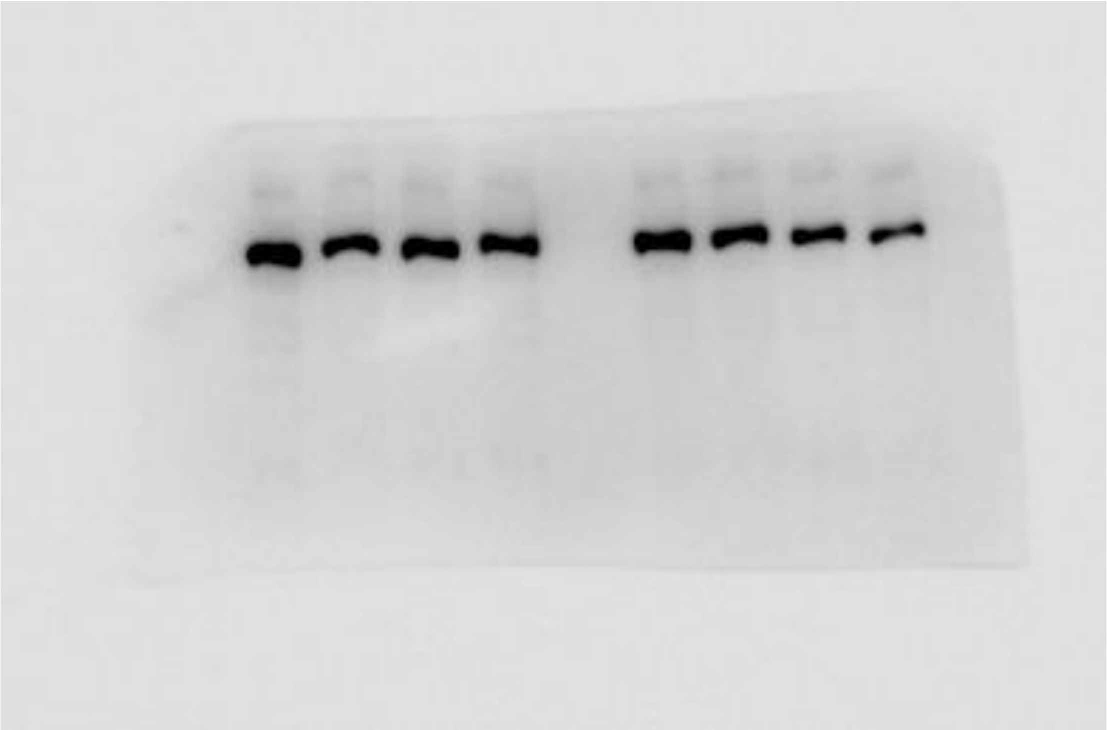

150 nm SiNP

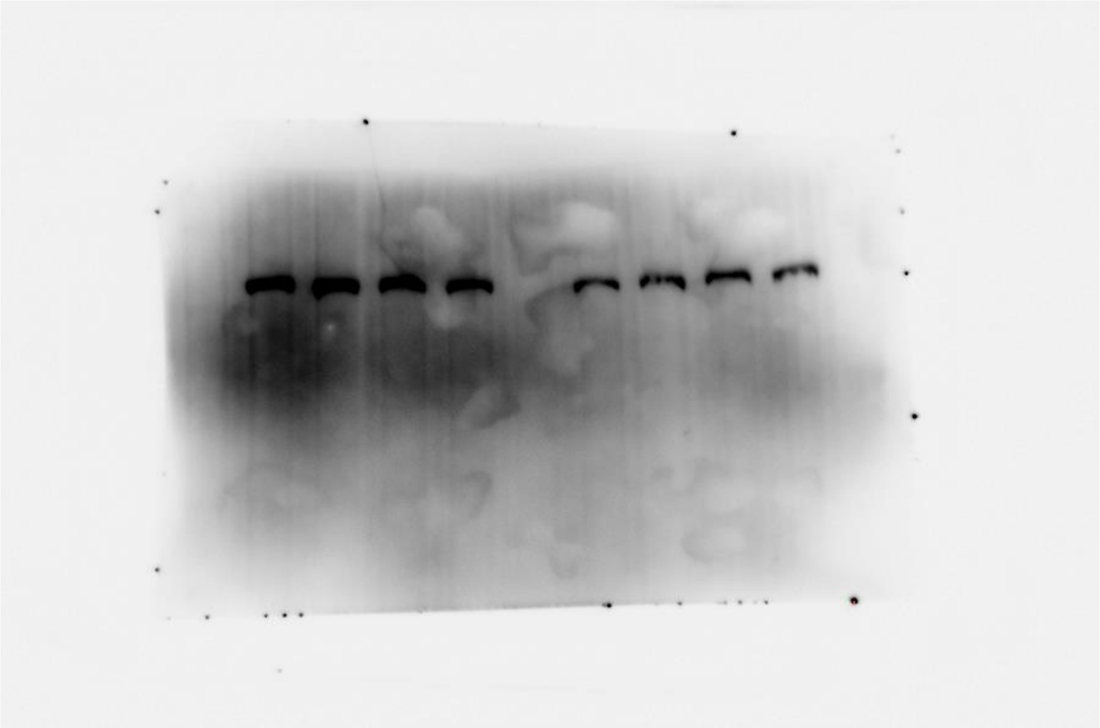

LC3A/B

- 50 nm SiNP and 100 nm SiNPs

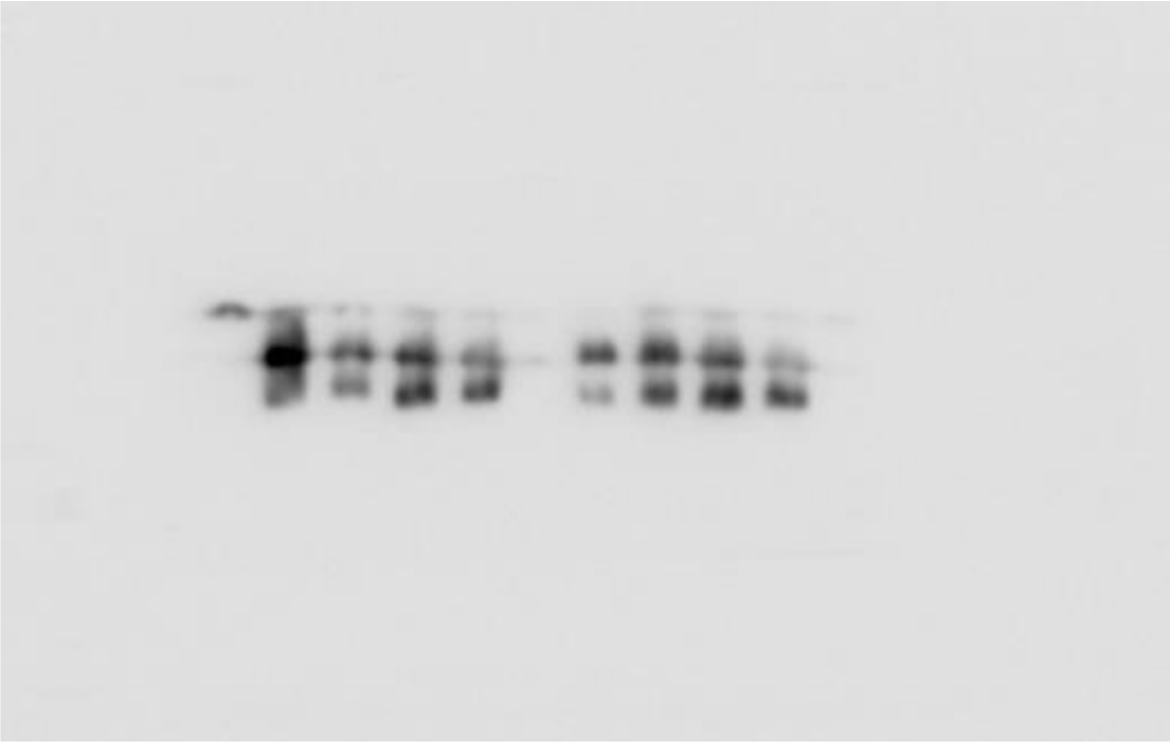

150 nm SiNP

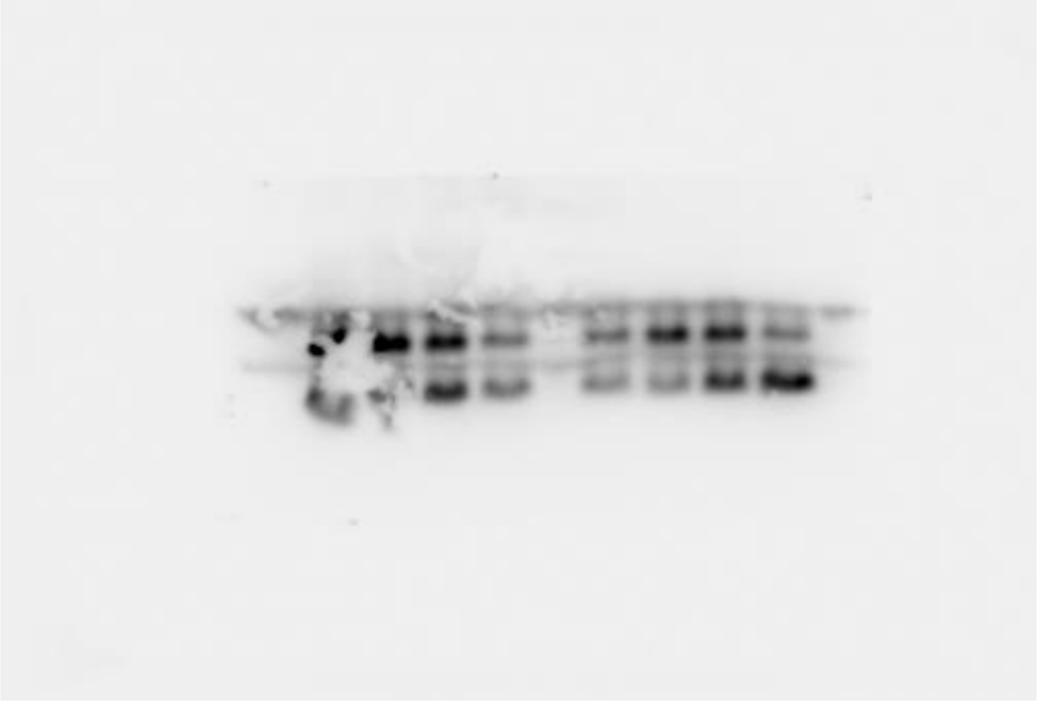

actin

50 nm SiNP

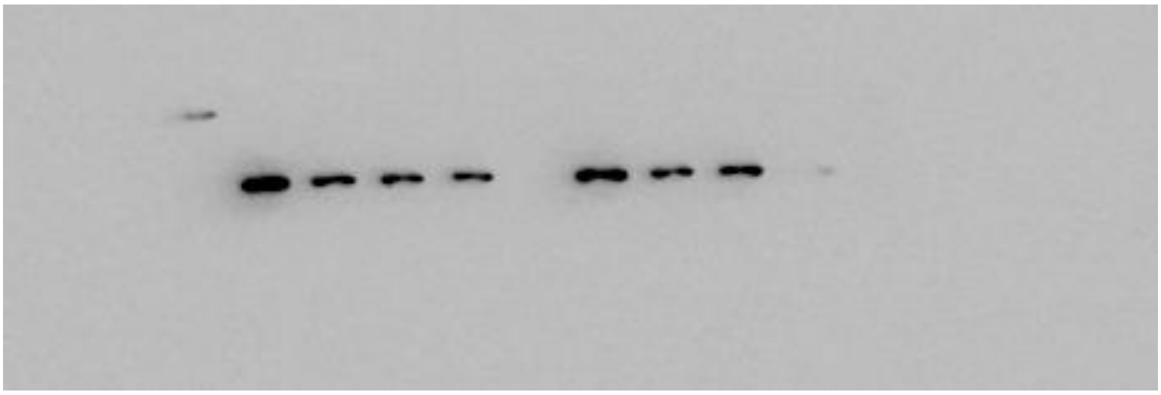

100 nm SiNP

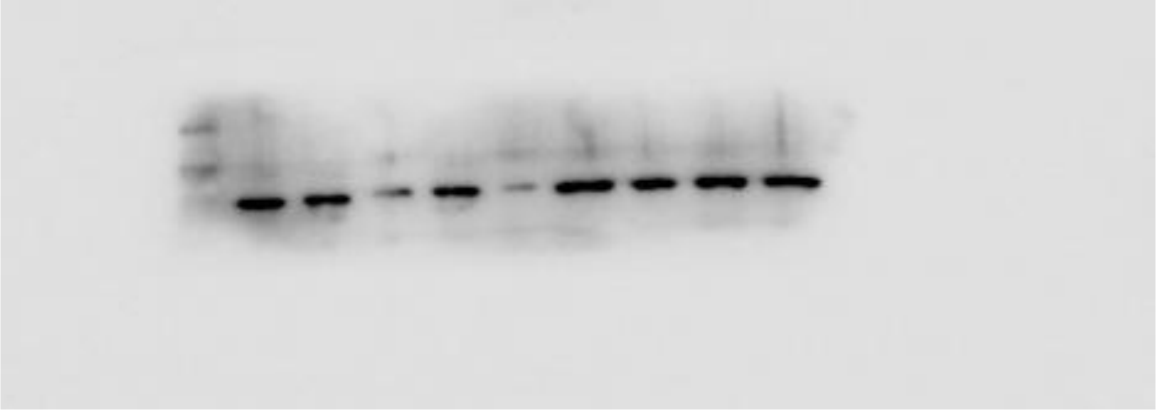

150 nm SiNP

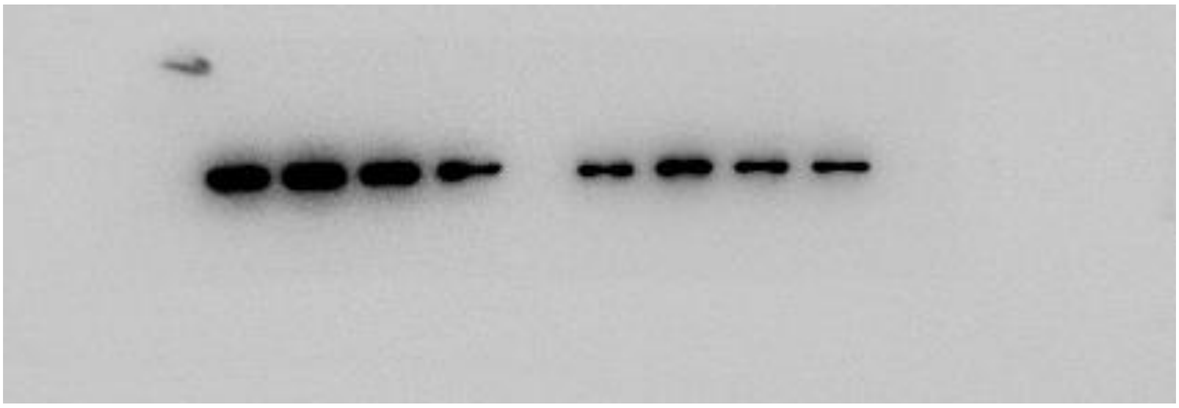

Supplementary figure 1. The acute cytotoxicity induced by SiNPs treatment in R28, retinal precursor cell line.

A) Flow cytometric analysis after TUNEL staining. R28 cells were treated with 50, 100, 150 nm sized SiNPs at different concentrations (0 to 50 μg/ml) for 24 hours. Increased TUNEL positive populations (green in color) were observed in high concentrations of 100 and 150 nm SiNPs treatment. **B)** Representative TEM image of SiNPs induced toxicity in R28 cells. Compared to the normal control with intact mitochondria, SiNPs treated cells show SiNPs accumulation in cytoplasmic vesicles and necrosis (arrowhead).

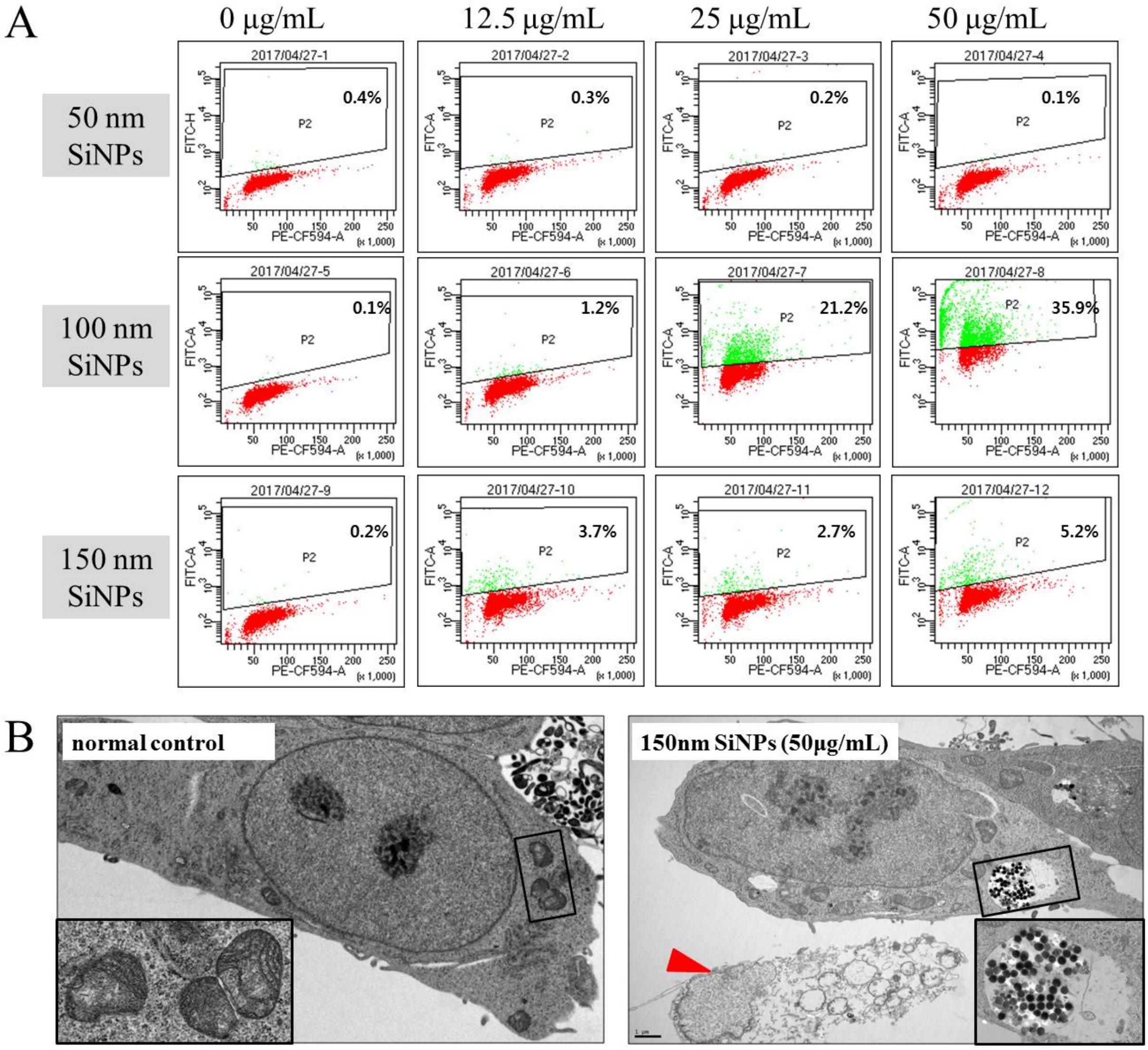

Supplement: Supplementary file 1 — supplementary material [file 41598_2017_15247_MOESM1_ESM.pdf]
